# Uterine artery Doppler in the management of early pregnancy loss: a prospective, longitudinal study

**DOI:** 10.1186/s12884-015-0464-9

**Published:** 2015-02-13

**Authors:** Luís Guedes-Martins, Joaquim P Saraiva, Ana R Gaio, Ana Reynolds, Filipe Macedo, Henrique Almeida

**Affiliations:** Department of Experimental Biology, Faculty of Medicine, University of Porto, 4200-319 Porto, Portugal; Hospital Centre of Porto EPE, Department of Women and Reproductive Medicine, Largo Prof. Abel Salazar, 4099-001 Porto, Portugal; Obstetrics-Gynecology, Private Hospital Trofa, 4785-409 Trofa, Portugal; Department of Mathematics, Faculty of Sciences, University of Porto, 4169-007 Porto, Portugal; CMUP-Centre of Mathematics, University of Porto, 4169-007 Porto, Portugal; Centro de Simulação Médica do Porto (CESIMED), 4465-024 São Mamede de Infesta, Portugal; Department of Medicine, Faculty of Medicine, University of Porto, 4200-319 Porto, Portugal; Department of Cardiology, S. João Hospital Centre, 4200-319 Porto, Portugal; Obstetrics-Gynecology, CUF-Hospital Porto, 4100 180 Porto, Portugal

**Keywords:** Early pregnancy loss, Incomplete miscarriage, Uterine artery Doppler

## Abstract

**Background:**

The pharmacological management of early pregnancy loss reduced substantially the need for dilation and curettage. However, prognostic markers of successful outcome were not established. Thus the major purpose of this study was to determine the sensitivity and specificity of the uterine artery pulsatility (PI) and resistance (RI) indices to detect early pregnancy loss patients requiring dilation and curettage after unsuccessful management.

**Methods:**

A cohort prospective observational study was undertaken to include women with early pregnancy loss, ≤ 12 weeks of gestation, managed with mifepristone (200 mg) and misoprostol (1600 μg) followed by PI and RI evaluation of both uterine arteries 2 weeks after. At this time, in 173/315 patients, incomplete miscarriage was diagnosed. Among them, 32 underwent uterine dilatation and curettage at 8 weeks of follow-up.

**Results:**

The cut-off points for the uterine artery PI and RI, leading to the maximum values of sensitivity (69.5%, CI_95%_: 61.5%-76.5% and 75.0%, CI_95%_: 57.9%-86.8%, respectively) and specificity (75.0%, CI_95%_: 57.9%-86.8% and 65.6%, CI_95%_: 48.3%-79.6%, respectively), for the discrimination between the women who needed curettage from those who resolved spontaneously were 2.8 and 1, respectively.

**Conclusions:**

The potential usefulness of uterine artery Doppler evaluation to predict the need for uterine curettage in patients submitted to medical treatment for early pregnancy loss was demonstrated.

## Background

Spontaneous abortion, or miscarriage, has been defined as clinically recognized pregnancy loss before the 20^th^ week of gestation [1], and 80% of these events occur in the first 12 weeks [2].

Although medical management with misoprostol, as an alternative to uterine dilation and curettage (D&C), has been associated with very high success rates of up to 99% in cases of early pregnancy loss [3-6], expectant management has also been indicated as a safe option for women with a first-trimester miscarriage [7-9]. The clear benefits of each of these approaches compared to the others have not been demonstrated [4,8], and management decisions have been adapted to the local circumstances and characteristics of patients.

The lack of consensus on the criteria for the success or failure of medical management in early miscarriage [4,7-10] precludes comprehensive conclusions regarding the obtained results. In the clinical setting, this lack of comparative data hinders a timely decision regarding whether to proceed with a uterine D&C, which, although it is a simple procedure, could result in serious complications. Clinical markers are thus needed to reliably support conservative management of early pregnancy loss [8,9,11].

Although uterine sonographic assessment in incomplete miscarriages might estimate the amount of ovular tissue remaining in the uterine cavity and the decidua thickness, the data fail to accurately predict the outcome when conservative management is an option [7]. More recently, a color Doppler evaluation of the uterine cavity contents was proposed to explore persistent communication between the residual trophoblasts and maternal circulation [11]; in contrast to the simple assessment of retained material volume, the presence or absence of flow were able to provide a quantitative estimate of the success of conservative management. Based on these findings, we reasoned that the extension of communication to the uterine artery would produce additional measurable Doppler changes in its impedance. In this case, the changes would enhance the predictability of successful conservative management and decrease the need for D&C.

The main purpose of this study was to determine the sensitivity and specificity of the uterine artery pulsatility (PI) and resistance (RI) indices, aiming to detect the patients with early pregnancy loss requiring D&C after unsuccessful conservative management using mifepristone *plus* misoprostol.

## Methods

### Study population and design

The research protocol was approved by the local ethics committee [IRB protocol number: 150/13(096-DEFI/122-CES)] of Hospital Center of Porto (CHP). All of the subjects gave their informed consent.

This is a cohort prospective observational study including women with early pregnancy loss (≤12 weeks gestation), as determined by the last menstrual period, attended at the emergency department of a tertiary university hospital center (CHP), from January 2011 to March 2013.

The diagnosis of early pregnancy loss was confirmed by vaginal ultrasound based on the guidelines of the Royal College of Obstetricians and Gynaecologists in the UK.^12^ The vaginal ultrasound criteria for early miscarriage-empty sac are an intra-uterine image consistent with a gestational sac with a mean diameter > 20 mm with no detectable embryonic pole or if the diameter is ≤ 20 mm with no change on rescan at 7 days. The criteria for early miscarriage-missed miscarriage are the visualization of a gestational sac with an embryo/fetus with a crown-rump length (CRL) > 6 mm with no heart activity or ≤ 6 mm with no change on rescan 7 days later [12]. Criteria for complete miscarriage included no evidence of retained products of conception in the uterine cavity and endometrial thickness (ET) < 12 mm (previous work [7] used ET < 15 mm). The sonographic criteria for incomplete miscarriage were: presence of heterogeneous features in the uterine cavity (without gestational sac) and endometrial midline echo distortion (at any endometrial thickness).

The inclusion criteria were cases of early pregnancy loss, defined as intrauterine pregnancy with reproducible evidence of lost fetal heart activity and/or the lack of increased crown–rump length over one week or the persisting presence of an empty sac at less than 12 weeks of gestation [10] (empty sac or missed miscarriage) in clinically stable women. The exclusion criteria included ectopic pregnancy, complete or incomplete miscarriage, known allergy to prostaglandins or NSAIDS, multiple gestation, heavy vaginal bleeding or hemodynamic instability, blood clotting problems or current treatment with anticoagulants, hemoglobin < 10 g/dL, body temperature > 38°C, CRL larger than 12 weeks of gestation and prostaglandin contraindications, including uncontrolled blood pressure, mitral stenosis, severe asthma or glaucoma.

### Management protocol, data collection and follow-up

#### Phase 1 Management

For each patient, the last menstrual period, age, parity, educational level, previous history of spontaneous first trimester miscarriage, body mass index, history of smoking, and previous uterine curettage were recorded.

Upon diagnosis of early pregnancy loss, confirmed by an experienced sonographer, a management protocol adopted in the department was employed. Patients with no or light vaginal bleeding were medicated with 200 mg of mifepristone [13] orally and followed-up as outpatients. They were instructed to make an additional appointment 48 h later or earlier in case of severe pain, bleeding or fever. At this time, unless the uterus was empty, the patients were admitted to the hospital and treated with 1600 μg of vaginal misoprostol divided into two doses, administered 4 hours apart. Eight hours after starting the procedure, a uterine ultrasound exam was performed followed by D&C in the cases in which the gestational sac was evident. The stable patients without evidence of a gestational sac were discharged and enrolled in Phase 2.

#### Phase 2 Management

At the end of the second week following the completed diagnosis, the patients visited the hospital for a review of the symptoms and a pelvic sonogram, including a uterine artery Doppler study. The patients were divided into three groups (I, II and III). When the condition was considered resolved (complete miscarriage), the patients were assigned to group I. In the cases of incomplete miscarriage, the patients were required to revisit the hospital 6 weeks later (8 weeks after the initial diagnosis). At this time, if resolution had been achieved (complete miscarriage), the patients were assigned to group II, whereas patients with a persistence of an incomplete miscarriage, were assigned to group III and submitted to D&C.

### Doppler flow assessment

**Figure 1:**
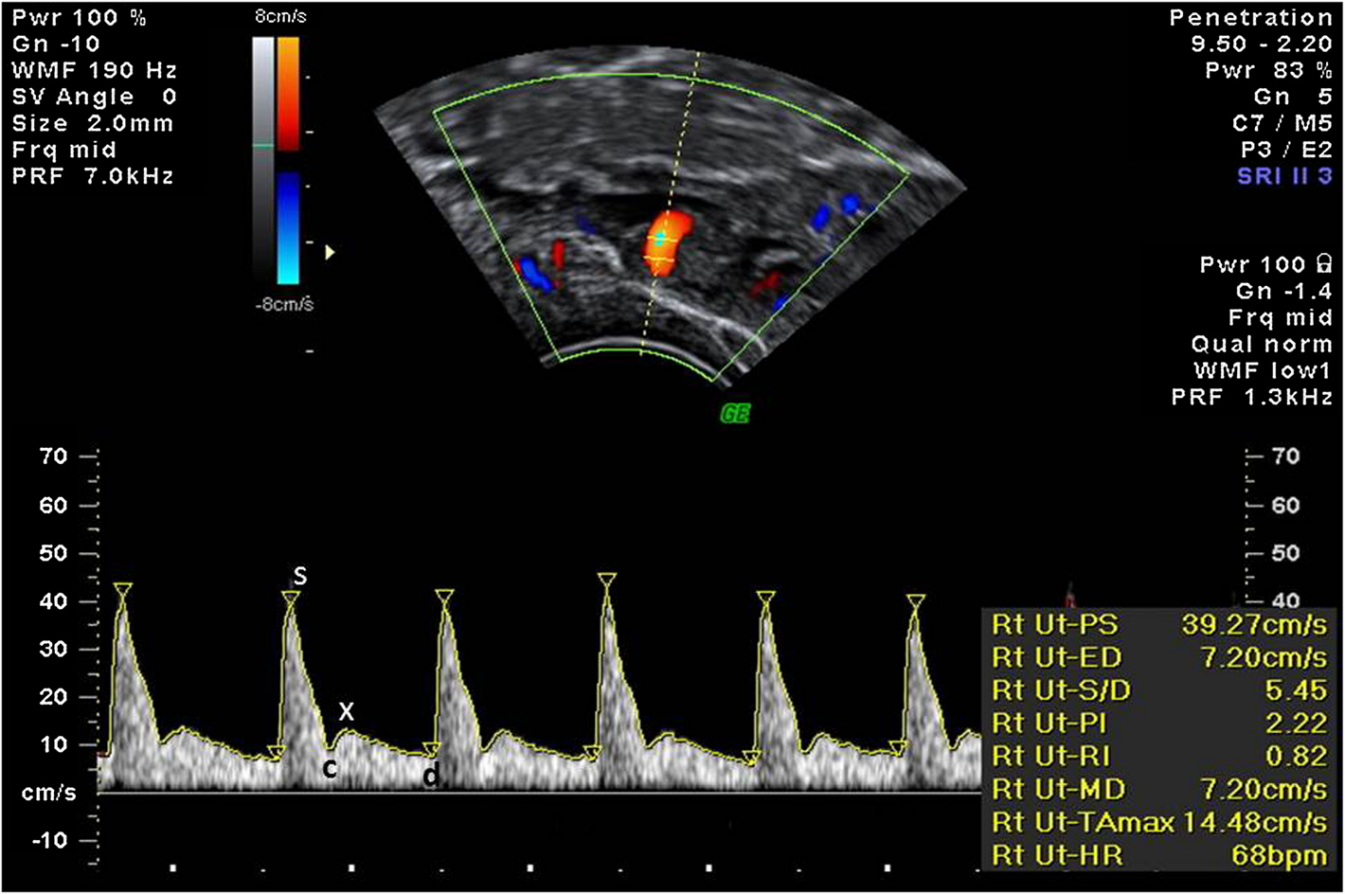
**Doppler spectra of uterine artery flow.** Pulsatility index (PI) is used as a measure of impedance of the flow of blood distal to the sampling point and is automatically calculated according to the formula $PI=\frac{\left(s-d\right)}{mean}$ where *s* is the peak *d* is the minimum and the average is the *mean* maximum Doppler shift frequency over the cardiac cycle. Resistance index (RI) is automatically calculated using the formula $RI=\frac{\left(s-d\right)}{s}$ *s*, peak systolic; *d*, end-diastolic; *c*, early diastolic; *x*, maximum diastolic frequency.

At the 2^nd^ week of follow-up uterine artery (UtA) Doppler assessment was performed by a single, experienced operator (L.G-M, 6 years of experience in obstetric and gynaecologic ultrasound) to avoid inter-observer variability. A Voluson 730 Pro (GE Healthcare Technologies, Milwaukee, WI, USA) multifrequency transvaginal and transabdominal ultrasound device equipped with a 5 MHz transvaginal transducer was employed. A sagittal plane of the uterus including the viewing of the cervical canal and internal cervical os was obtained. Subsequently, the transducer was gently tilted from side to side and each uterine artery was identified at the level of the internal os with the aid of a color flow mapping. Pulsed wave Doppler was used with a sampling gate set at 2 mm to cover the entire vessel, ensuring that the angle of insonation was less than 30°. When three similar consecutive waveforms were obtained - Figure 1 - mean PI and RI of the left and right arteries were calculated. The presence or absence of a bilateral early protodiastolic notch in the UtA was noticed. A positive notch was defined as a persistent decrease in the blood flow velocity in the early diastole, below the diastolic peak velocity in at least one UtA Doppler ultrasound spectrum. Notch absence was defined by its bilateral absence.

The intra-observer reliability was obtained from two readings, at the beginning and the end of the scan, on the first 33 recordings of the resistance and pulsatility indices in the uterine arteries. The intraclass correlation coefficients (ICC) and 95% confidence intervals were calculated using a two-way mixed-effects model with absolute agreement. The reliability coefficient, which is the difference value that will be exceed by only 5% of the pairs of measurements in the identical subject, was calculated as 1.96 times the standard deviation of the difference between the pairs of repeated measurements.

### Statistical analysis

The statistical inference from the descriptive measures comprised the chi-squared test or Fisher’s test (as adequate) to evaluate the fitting of a categorical variable distribution to the uniform distribution, and the Wilcoxon rank sum test to compare the medians from two independent populations. The Kruskal-Wallis test (or ANOVA on ranks) was used to assess whether samples from different groups originate from the same population. The pairwise comparisons between the group levels used the Wilcoxon rank sum test with corrections for multiple testing following Holm’s method [14]. Non-parametric techniques were applied once the normality and homoscedasticity of the residuals from parametric models were not satisfied.

Receiver-operating characteristic (ROC) curves evaluated the discrimination ability of the RI and PI, independently and between the women who underwent curettage and those who had a spontaneous resolution of the clinical condition. These curves plot the true positive rate (Sensitivity) against the false positive rate (1-Specificity) at various discrimination threshold settings. The closer the ROC curve is to the upper left corner, the higher the overall accuracy of the test, and this criterion was used to identify the optimal thresholds [15]. At these cut-off points, the likelihood ratios (LRs) for the positive and negative tests (the LR (+ve test) and the LR (−ve test), respectively) and the positive and negative predicted values were computed. In particular, the pretest odds for D&C multiplied by the LR (+ve test) or the LR (−ve test) determines the post- (+ve test) or post- (−ve test) odds, respectively. The positive tests are associated with D&C if the LR (+ve test) is greater than 1, and the negative tests are associated with no D&C if the LR (−ve test) is smaller than 1. The further away the values are from 1, the stronger the evidence [16].

All of the statistical analyses were carried out using the R language and software environment for statistical computation, version 2.12.1. The significance level was fixed at 0.05.

## Results

**Figure 2:**
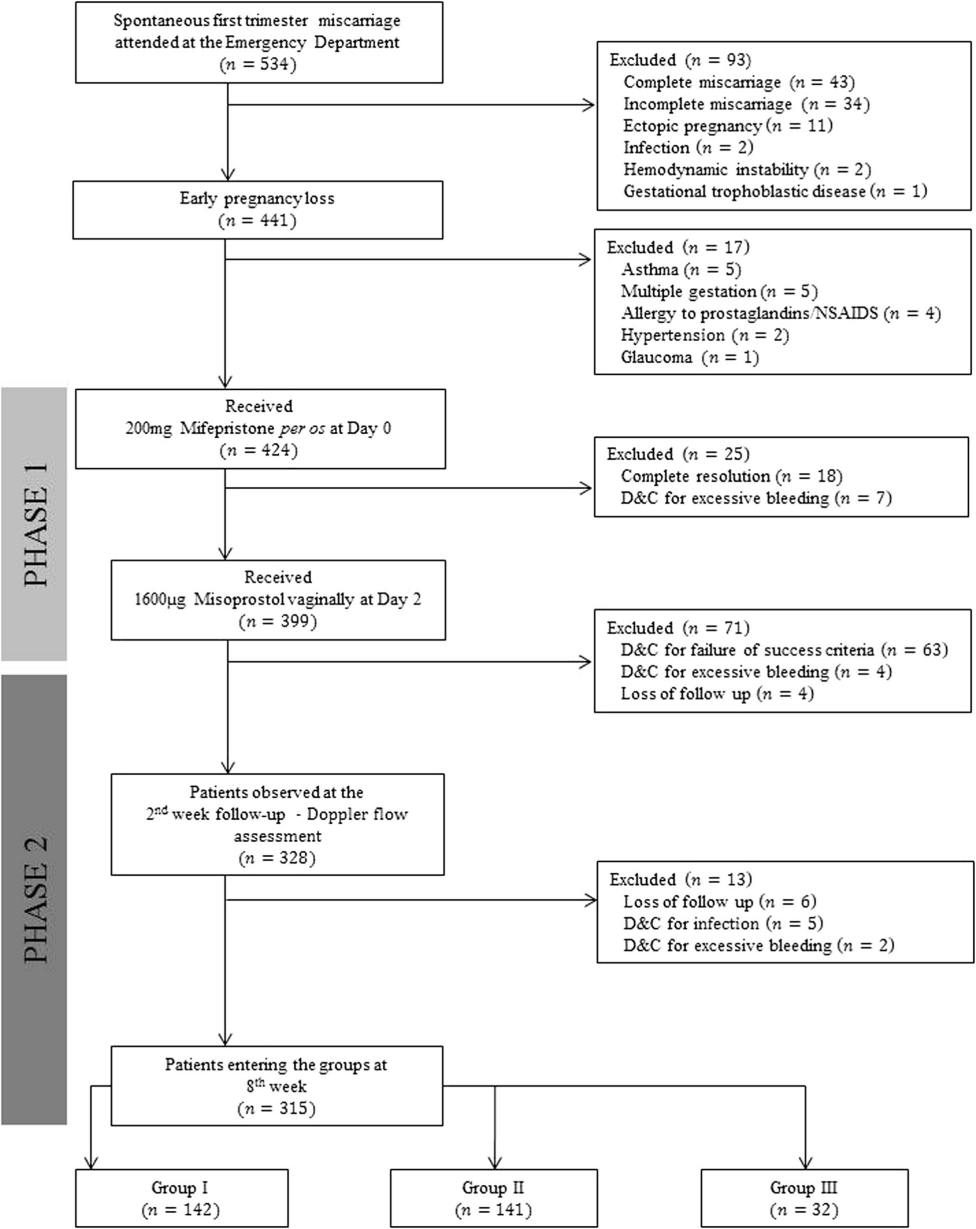
**Study flowchart: number of patients (n), follow-up, Doppler assessment and grouping.** Group I – complete miscarriage at the 2^nd^ week. Group II – incomplete miscarriage at the 2^nd^ week and complete miscarriage at the 8^th^ week. Group III – incomplete miscarriage at 2^nd^ and 8^th^ week of follow-up, submitted to D&C. D&C, dilation and curettage.

Of the 534 consecutive cases with spontaneous first trimester miscarriages who were attended at the emergency department during the study period, 315 (59%) patients entered the final groups, as shown in the flowchart in Figure 2.

Table 1 describes the patient demographic data and variables considered in the study, with the statistical analysis. As previously defined, groups I, II and III included 142 (45%), 141 (45%) and 32 (10%) patients, respectively. The histological examination of the intra-uterine contents after D&C in the group III patients (n = 32) revealed conception debris/ovular tissue in all cases. Only 1% of all the included cases showed an absence of bilateral notching on the Doppler evaluation of the uterine artery blood flow.

**Table 1:** **The demographic data, uterine content thickness and Doppler assessment in all groups and in each group (I, II and III) of the included patients**

		**All n(%) 315(100)**	**p-value** ^**1**^	**Group I n(%) 142(45)**	**p-value** ^**1**^	**Group II n(%) 141(45)**	**p-value** ^**1**^	**Group III n(%) 32(10)**	**p-value** ^**1**^
Age (in years)	20-24	36(12)	**<0.001**	18(13)	**<0.001**	14(10)	**<0.001**	4(12)	0.030
	25-34	159(50)		67(47)		76(54)		16(50)	
	35-46	120(38)		57(40)		51(36)		12(38)	
Education level (in years)	≤9	115(37)	**<0.001**	55(39)	**<0.001**	48(34)	**<0.001**	12(38)	0.197
	10-12	146(46)		60(42)		72(51)		14(44)	
	>12	54(17)		27(19)		21(15)		6(19)	
Parity	0	171(54)	0.128	64(45)	0.240	49(49)	0.842	25(78)	**0.001**
	≥1	144(46)		78(55)		51(51)		7(22)	
Previous spontaneous first trimester miscarriage (≤12 weeks)	1	277(88)	**<0.001**	125(88)	**<0.001**	124(88)	**<0.001**	28(88)	**<0.001**
	>1	38(12)		17(12)		17(12)		4(12)	
Body Mass Index (Kg/m^2^)	16-24	142(45)	**<0.001**	72(51)	**<0.001**	53(38)	**0.001**	17(53)	**0.001**
	25-29	134(43)		58(41)		62(44)		14(44)	
	30-44	39(12)		12(8)		26(18)		1(3)	
Smoking	No	243(77)	**<0.001**	103(39)	**<0.001**	115(82)	**<0.001**	25(78)	**0.001**
	Yes	72(23)		73(27)		26(18)		7(22)	
GA at early pregnancy loss diagnosis [weeks (SD)]		8.0(1.3)		7.6(1.0)		8.2(1.3)		9.1(1.3)	
Uterine arteries Doppler assessment [days (SD)]		18.2(2.8)		18.0(2.4)		18.3(3.2)		18.9(2.6)	
Intra-uterine content thickness [mm (SD)]		11.8(5.8)		6.1(2.5)		15.8(2.3)		19.6(2.3)	
Uterine content thickness at 2^nd^ week follow-up visit	<12 mm	148(0.53)	0.328	142(100)		0		0	
	≥12 mm	167(0.47)		0		141(100)		32(100)	
Uterine artery Doppler - Bilateral notching	No	3(1)	**<0.001**	0	**<0.001**	1(1)	**<0.001**	2(6)	**<0.001**
	Yes	312(99)		142(100)		140(99)		30(94)	
Decision for D&C at 8^th^ week follow-up visit	No	283(90)	**<0.001**	142(100)		141(100)		0	
	Yes	32(10)		0		0		32(100)	

Legend: GA-gestational age; D&C - Dilation and Curettage; ^1^Chi-square test or Fisher’s test, as adequate; Statistical analysis (p) was obtained to evaluate the differences within each demographic and clinical characteristics.

For the Doppler indices (PI and RI), the null hypothesis that samples from each group of patients (I, II and III) originate from an identical population could be rejected (Table 2).

**Table 2:** **Comparison of the average (min-max) pulsatility (PI) and resistance (RI) indices in the uterine arteries between groups (I + II) and III**

	**Group I + II (** ***n = 283*** **)**	**Group III (** ***n = 32*** **)**	**p-value** ^**1**^
PI	3.55(1.17-8.01)	2.57(1.16-3.97)	<0.001
RI	1.00(0.66-1.00)	0.90(0.75-1.00)	<0.001

^1^Wilcoxon rank sum test with corrections for multiple testing following the Holm’s method.

The reliability coefficients were 0.127 and 0.024 for the PI and RI, respectively. The ICC values for the evaluation of the intraobserver reliability concerning the PI and RI measurements were very high, at 0.999 (range: 0.998 to 1.000) and 0.996 (range: 0.991 to 0.998), respectively.

**Figure 3:**
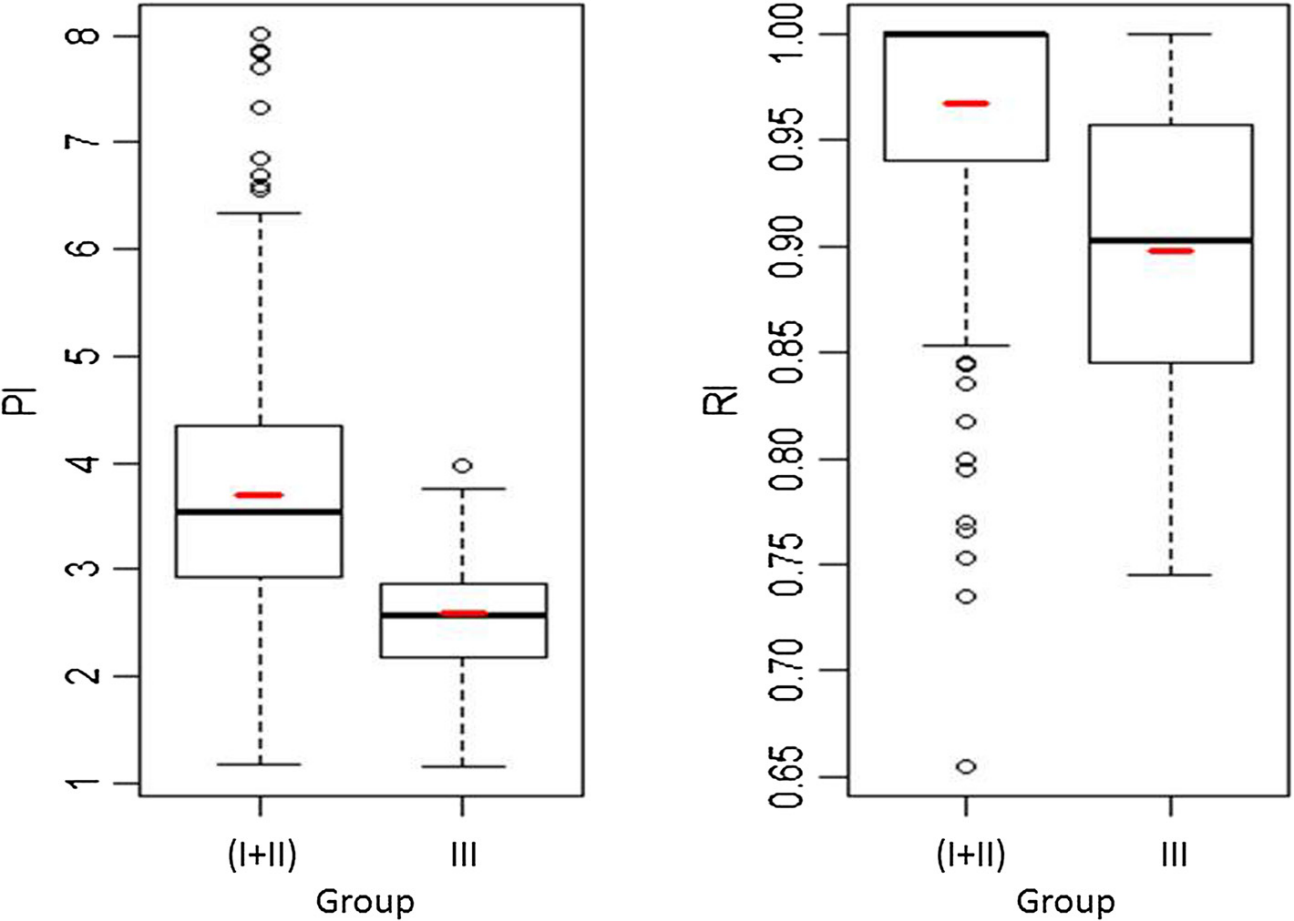
**The boxplot of the pulsatility (PI) and resistance (RI) indices for the patients in groups I + II (n = 283) and III (n = 32).** The mean of each boxplot is marked with a small horizontal line.

**Figure 4:**
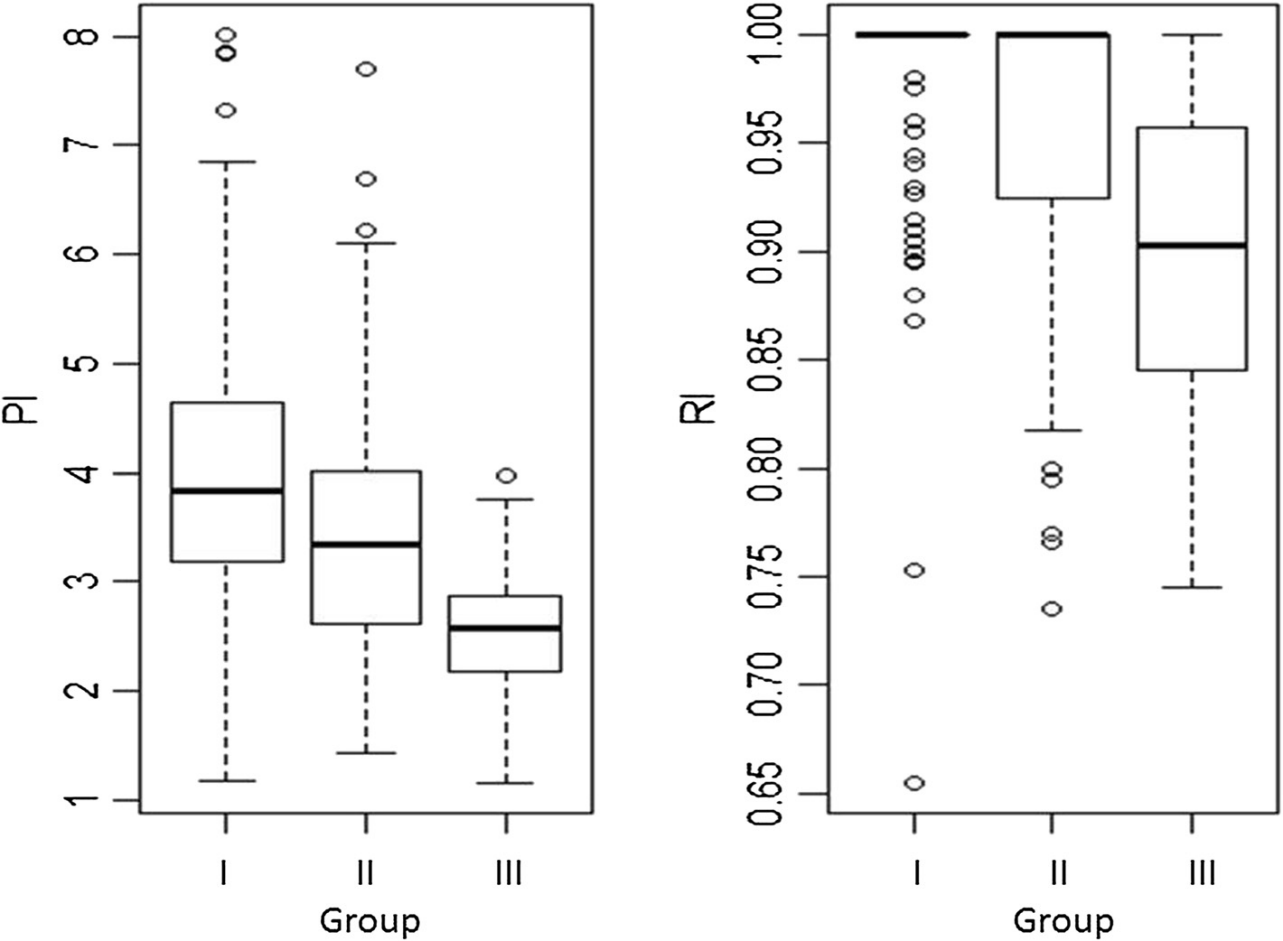
**The boxplot for pulsatility (PI) and resistance (RI) indices according to each studied group.**

Table 3 presents the average and the variation (minimum – maximum) of the Doppler indices obtained in each studied group. The non-parametric Kruskal-Wallis test (ANOVA on ranks; IR: chi-squared = 60.331, df = 2, p-value < 0.001; IP: chi-squared = 50.610, df = 2, p-value < 0.001) was used, followed by Holm’s method for multiple comparisons. Within each index, all three pairwise comparisons were statistically significant (p < 0.001) – Figures 3 and 4.

**Table 3:** **The average (min-max) pulsatility (PI) and resistance (RI) indices of the uterine arteries in groups I, II and III**

	**Group I (** ***n = 142*** **)**	**Group II (** ***n = 141*** **)**	**Group III (** ***n = 32*** **)**
PI	3.84 (1.17-8.01)	3.35 (1.43-7.70)	2.57 (1.16-3.97)
RI	1.00 (0.66-1.00)	1.00 (0.74-1.00)	0.90 (0.75-1.00)

**Figure 5:**
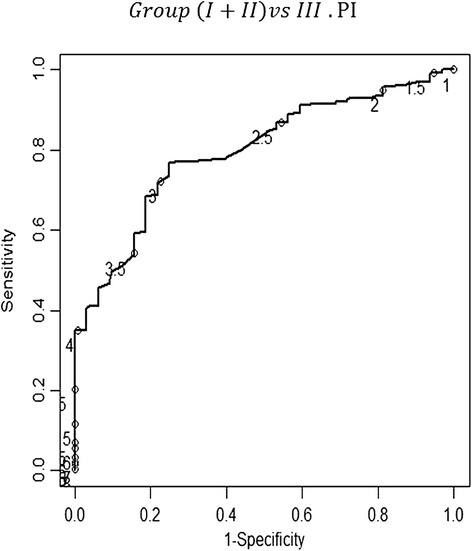
**Receiver-operator characteristic (ROC) curve for the different values of the pulsatility index concerning the discrimination between groups (I + II) and III.** The curve describes the association between sensitivity and specificity at different cut-offs of the pulsatility index. Values from 1 to 8 in intervals of 0.5 are identified with a circle. The point in the curve that is closer to the upper-left corner of the plot corresponds to the value 2.78 of the pulsatility index. At that cut-off point, the sensitivity and specificity values of the pulsatility index in the uterine artery were 77.0% (CI _95%_: 71.8%-81.6%) and 75.0% (CI _95%_: 57.9%-86.8%), respectively; the area under the curve (AUC) was 0.796 (0.727-0.865). The correspondent positive and negative predictive values were 96.5% and 27.0%, respectively. The likelihood ratio (LR) for a positive or negative test was 3.08 (CI _95%_: 1.68%-5.63%) or 0.31 (CI _95%_: 0.23%-0.41%), respectively.

**Figure 6:**
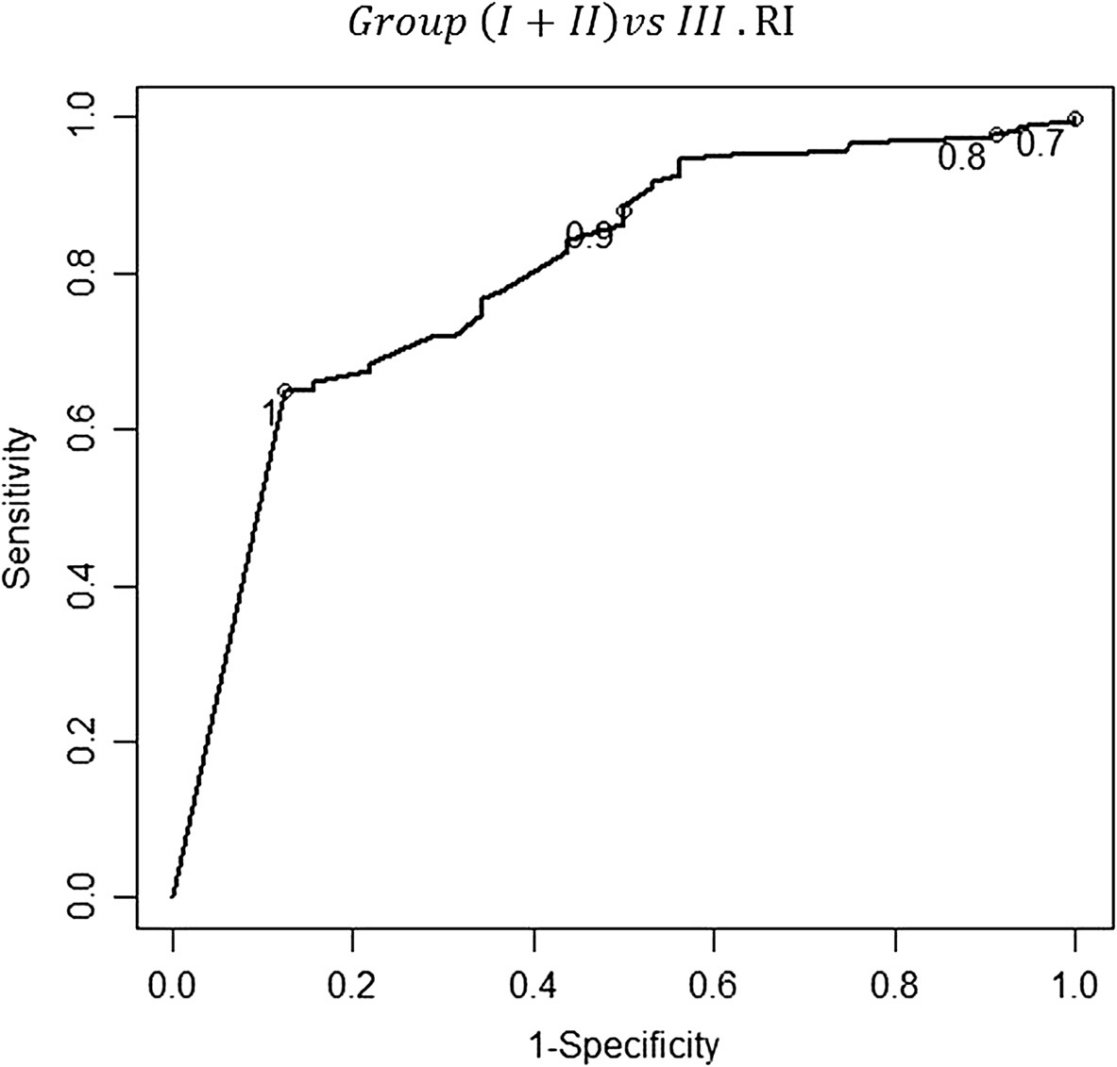
**The receiver-operator characteristic (ROC) curve for the different values of the resistance index concerning the discrimination between groups (I + II) and III.** The values 0.7, 0.8, 0.9 and 1.0 are identified in the curve with a circle. The point in the curve that is closer to the upper-left corner of the plot corresponds to value 1 for the resistance index. At that cut-off point, the sensitivity and specificity values of the resistance index in the uterine artery were of 65.0% (CI _95%_: 59.3%-70.3%) and 87.5% (CI _95%_: 71.9%-95.0%), respectively; the area under the curve (AUC) was 0.798 (0.754-0.843). The correspondent positive and negative predictive values were 97.9% and 22.0%, respectively. The likelihood ratio (LR) for a positive or negative test was 5.20 (CI _95%_: 2.07%-13.06%) or 0.40 (CI _95%_: 0.33%-0.49%), respectively.

The Doppler of the uterine arteries was first used to discriminate the women in group III from those in groups I + II. The cut-off points for the uterine artery pulsatility (PI) and resistance (RI) indices, providing the maximum values of sensitivity (77.0%, CI_95%_: 71.8%-81.6% and 65.0%, CI_95%_: 59.3%-70.3%, respectively) and specificity (75.0%, CI_95%_:57.9%-86.8% and 87.5%, CI_95%_: 71.9%-95.0%, respectively) were 2.78 and 1, respectively, for distinguishing the women in group III from those in groups I + II. A test was considered positive if the PI or RI was less than 2.78 or 1, respectively. The positive predictive values were 96.5% and 97.9% for the PI and RI, respectively. The negative predictive values were 27.0% and 22.0% for the PI and the RI, respectively. The LR for a positive or negative test was 3.08 (CI _95%_: 1.68%-5.63%) or 0.31 (CI _95%_: 0.23%-0.41%), respectively, for the PI. Similarly, the LR for a positive or negative test was 5.20 (CI _95%_: 2.07%-13.06%) or 0.40 (CI _95%_: 0.33%-0.49%), respectively, for the RI – Figures 5 and 6.

**Figure 7:**
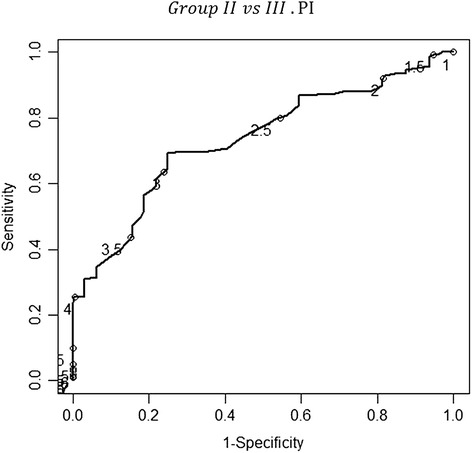
**The receiver-operator characteristic (ROC) curve for the different values of the pulsatility index concerning the discrimination between groups II and III.** All the values from 1 to 8 in increments of 0.5 are identified in the curve with a circle. The point in the curve that is closer to the upper-left corner of the plot corresponds to the value 2.808 of the pulsatility index. At that cut-off point, the sensitivity and specificity values of the pulsatility index in the uterine artery were of 69.5%( CI _95%_: 61.5%-76.5%) and 75.0%( CI _95%_: 57.9%-86.8%), respectively; the area under the curve (AUC) was 0.734 (0.648-0.819). The correspondent positive and negative predictive values were 92.5% and 35.8%, respectively. The likelihood ratio (LR) for a positive or negative test was 2.78 (CI _95%_: 1.51%-5.12%) or 0.41 (CI _95%_: 0.30%-0.56%), respectively.

**Figure 8:**
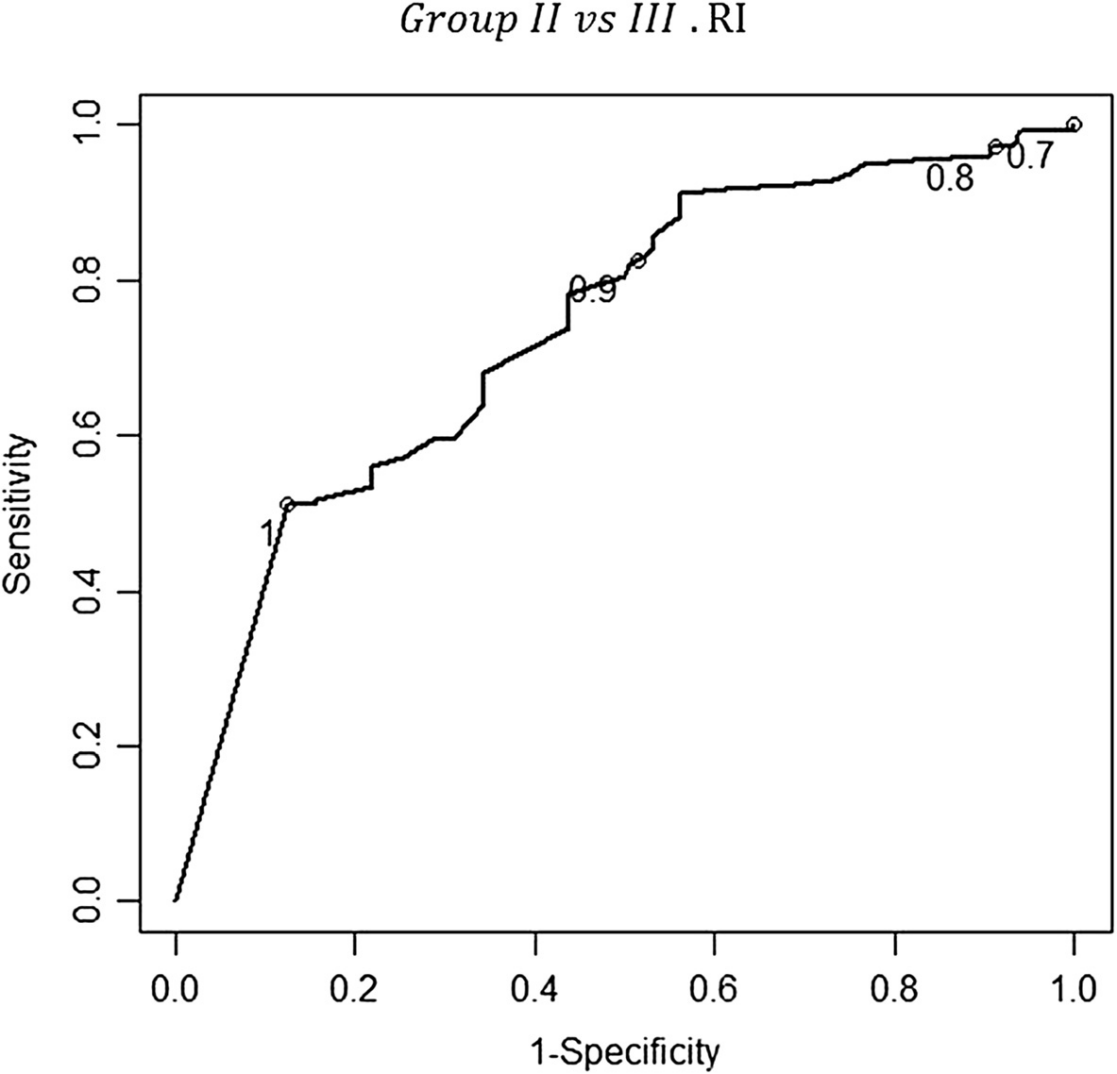
**The receiver-operator characteristic (ROC) curve for the different values of the resistance index concerning the discrimination between groups II and III.** The values 0.7, 0.8, 0.9 and 1.0 are identified in the curve with a circle. The point in the curve that is closer to the upper-left corner of the plot corresponds to a value of 1 for the resistance index. At that cut-off point, the sensitivity and specificity values of the resistance index in the uterine artery were of 75% (CI _95%_: 57.9%-86.8%) and 65.6% (48.3%-79.6%), respectively; the area under the curve (AUC) was 0.738 (0.676-0.799). The correspondent positive and negative predictive values were 89.9% and 32.8%, respectively. The likelihood ratio (LR) for a positive or negative test was 2.18 (CI _95%_: 1.34%-3.55%) or 0.38 (CI _95%_: 0.26%-0.56%), respectively.

Additionally, uterine artery Doppler was used to discriminate between the women in group III and those in group II. This discrimination was the main goal of the study. The cut-off points for the uterine artery pulsatility (PI) and resistance (RI) indices, with maximum values of sensitivity (69.5%, CI_95%_: 61.5%-76.5% and 75.0%, CI_95%_: 57.9%-86.8%, respectively) and specificity (75.0%, CI_95%_: 57.9%-86.8% and 65.6%, CI_95%:_ 48.3%-79.6%, respectively) were 2.80 and 1, respectively, for distinguishing the women in group III from those in group II. A test was considered positive if the PI or RI was less than 2.8 or 1, respectively, and negative otherwise. The positive predictive values were 92.5% and 89.9% for the PI and the RI, respectively. The negative predictive values were 35.8 and 32.8% for the PI and the RI, respectively. The LR for a positive or negative test was 2.78 (CI _95%_: 1.51%-5.12%) or 0.41 (CI _95%_: 0.30%-0.56%), respectively, for the PI. Similarly, the LR for a positive or negative test was 2.18 (CI _95%_: 1.34%-3.55%) or 0.38 (CI _95%_: 0.26%-0.56%), respectively, for the RI – Figures 7, 8.

## Discussion

In recent decades, the need for uterine evacuation in cases of early pregnancy loss (delayed miscarriages or incomplete miscarriages) has been a matter of debate. Because surgical procedures have potential risk and might result in complications, including infection, hemorrhage and uterine perforation, the decision to employ drugs or to adopt an expectant attitude are considered acceptable, safe and efficient. The success rate measured within a reasonable period of time, such as 2 weeks [9], did preclude the use of D&C. Success rates of 71% to 84% were reported for expectant attitudes [7,9,17], and, in cases in which misoprostol was administered, complete miscarriage was found in 83 to 86% of the patients for periods of follow up that spanned from 1 week up to 4 weeks [6,17-19]. The data indicate that within a reasonable period of time, the success rate is similar when the option is for the expectant attitude or the use of pharmacological means. However, a significant number of patients will have an unplanned admission to the hospital for surgery [8,17], and some patients will require an emergency D&C [9].

As accurate predictive criteria for successful conservative management of early pregnancy loss have not been established, new clinical indicators are necessary, particularly those that might predict the need for uterine surgical evacuation [8,11,18]. Considering that sonographic assessment of the uterine content volume in incomplete miscarriages did not accurately predict this need [7], other approaches must be devised.

Regarding the fundamental role of maternal-fetal circulation, a maternal pelvic blood flow study using Doppler ultrasound technology is useful in pregnancy follow-up [20,21]. As a consequence of the widespread availability of equipment, several studies have analyzed the uterine artery blood flow patterns in pregnant women, emphasizing the PI and RI. Both indices provide quantitative data on end-arteriolar impedance [22] and, therefore, on local vascular resistance and blood flow velocity.

The application of Doppler ultrasound in the study of the retained products of conception after miscarriage or delivery had been proposed [23,24], and its use in the assessment of incomplete early miscarriage was recently reported for a large number of patients [11]. It was demonstrated that the absence of blood flow in the remaining intra-uterine trophoblastic tissue is associated with a significantly higher success rate of expectant management when compared to the presence of flow [9,11].

In this investigation, we explored the likelihood that intra-uterine retained ovular products could modulate uterine artery blood flow and change the PI and RI. Our results indicate that this modulation does occur. Additionally, the data indicate that uterine artery low PI and RI persistence associates to a considerable risk [16] for uterine curettage 8 weeks after an incomplete early miscarriage diagnosis, a time when a spontaneous resolution is unlikely. At 2 weeks after the decision for expectant management, the indices were significantly lower in the cases of incomplete miscarriage, compared to those of complete miscarriage. At the first follow-up visit, Doppler findings allowed us to distinguish women who would achieve complete resolution (group I) from those who should remain under surveillance (group II + III). The UtA-RI and PI at 2 weeks showed a reasonable sensitivity, specificity and positive predictive value for the identification of those patients who would need a D&C 8 weeks after starting the treatment. The obtained LR values showed only a moderate usefulness of the UtA Doppler evaluation on the prediction of the need for D&C (due to incomplete miscarriage) in patients submitted to medical management for early pregnancy loss [16].

Finally, the reliability study demonstrated that the Doppler blood flow measurement of the PI-UtA and RI-UtA was highly repeatable by our sonographer [25]. We used the ICC to assess the repeatability because there is sufficient consensus in the scientific literature to consider that values for ICC > 0.7 reflect a very low measurement error [25,26].

### Study limitations and future research

The absolute values of the uterine artery PI and RI have not been validated for uneventful early pregnancy, representing a limitation of the study. Although the reference ranges for the uterine artery mean PI are well known, covering pregnancy from 6 to 41 weeks of gestation [21,27], further studies are required to establish the identical curves of normality in women with spontaneous abortion and managed expectantly. This reference range will be helpful for validating the use of uterine artery Doppler in the management of cases of early pregnancy loss.

## Conclusions

This is the first prospective study providing evidence that uterine artery Doppler evaluation can predict the need for D&C due to incomplete miscarriage, after management of early pregnancy loss using mifepristone plus misoprostol.

## Abbreviations

CHP: Hospital Center of Porto
CRL: Crown-rump length
D&C: Dilation and curettage
ET: Endometrial thickness
ICC: Intraclass correlation coefficients
LR: Likelihood ratio
PI: Pulsatility index
RI: Resistance index
ROC: Receiver-operating characteristic
UtA: Uterine artery
+/− ve: Positive/negative
